# The rendering of human phenotype and rare diseases in ICD-11

**DOI:** 10.1007/s10545-018-0172-5

**Published:** 2018-03-29

**Authors:** Christopher G. Chute

**Affiliations:** Johns Hopkins University, 2024 E. Monument, Suite 1-200, Baltimore, MD 21287 USA

## Abstract

ICD-11 (International Classification of Diseases, 11th Revision) is the next major revision of the ICD by the World Health Organization (WHO). ICD-11 differs dramatically from historical versions, as it is based on an underlying semantic network of terms and meaning, called the Foundation. To function as a mutually exclusive and exhaustive statistical classification, ICD-11 creates derivative linearizations from the network that is a monohierarchy with residual categories such as Not Elsewhere Classified. ICD-11 also introduces the widespread post-coordination of terms, which allows for highly expressive representation of detailed patient descriptions. Phenotyping features are included in many subchapters or the signs and symptoms chapter. Composite phenotype descriptions of specific presentations or syndromes can be represented though post-coordination. Rare diseases are well represented in the Foundation, though not all appear in the relatively shallow linearization hierarchies.

## Phenotype representation in ontologies

Conventionally, phenotype is the manifestation of findings in an individual attributable to genomic influences. Phenotyping, as is the case with many words, is being usurped by the clinical research community to mean an algorithm to identify like patients to define a study cohort; this is particularly so with the advent of electronic medical records, where these algorithms can be executed (Conway et al. 2011; Pathak et al. 2013; Mo et al. 2015). Debate flares as to whether phenotype is a discrete feature, such as an extra digit, or a comprehensive characterization of a clinical presentation as a constellation of features.

Regardless, an organized way to consistently describe clinical presentations and specific findings invokes the notion of a taxonomy, or some naming system. Previous work addresses some distinctions and the recent evolution of taxonomy vs. ontology (Cornet and Chute 2016). For the present purposes, ontologies are formal taxonomies rendered in an acyclic hierarchy that invoke a description logic (Baader 2003) (a computable subset of First-Order Predicate Logic) to assert relationships between and among terms. The use of description logics has advantages, such as OWL (Web Ontology Language [OWL]; World Wide Web Consortium [W3C] 2012b), as it permits reasoning across these terms and concepts, from simple subsumption to inferring that relationships should be instantiated even when they are not explicitly asserted.

Phenotyping features are discrete concepts that lend themselves well to ordered, ontological structures. It is obvious that one may want to group specific polydactylies under a common category, which, in turn, can be grouped under skeletal malformations. This is particularly evident in the elegant Human Phenotype Ontology (Kohler et al. 2017), which precisely orders such features using OWL predicates.

## Classifications

Classifications are not ontologies, nor are they intended to be. In their most simple form, a classification is a set of categories intended to aggregate a large number of things into a relatively smaller number of groups. Like ontologies, a classification may invoke an ordered hierarchy of categories, invoking increasingly specific category subtypes with each layer of the hierarchy. The connection between one level of a hierarchy and another is rarely specified by the logically formal “is-a” relationship, but more typically informal “broader-than” or “narrower-than” assertions; this is known as the Simple Knowledge Organization System (SKOS) (W3C 2012a) Classifications are not intended to support logical reasoning, though they can help to organize concepts and can have profound effects on technology and social attitudes (Bowker and Star 1999).

### Statistical classifications

Statistical classifications are a particular genre of classification that exhibit two properties:

1. All concepts in a statistical classification are mutually exclusive (one cannot count something more than once, since everything is prohibited from belonging to more than one category).
2. Statistical classifications are exhaustive (there is a place to put anything and everything in the domain of the classification).

These properties have their consequences, which are not always desirable; however, they are necessary to function predictably for statistically tabulating things.

The first property forces a peculiar behavior to any classification that invokes hierarchy to order its categories; specifically, such hierarchies must be monohierarchies (where any category must have one and only one parent). Using disease as an example, this implies that Gastric Cancer, which is a child of the Cancer category in most versions of the ICD, cannot be a child of the category Gastrointestinal Disease. On reflection, this makes sense if tabulations of morbidity or mortality categories are to sum to 100% and never exceed that. However, it is immediately clear that which category something that could belong to more than one category actually goes into can be arbitrary. In the case of the ICDs, such arbitrary distinctions derive from tradition or legacy; statisticians are loath to have statistics change from version to version of the ICD simply because a concept was placed into a new category. The rule of thumb for ICD-11 was to retain historical category assignments, unless there was compelling scientific evidence (such as gastric ulcers being in many cases now recognized as an infectious disease) to consider a new assignment.

The second property, that statistical classifications are exhaustive, raises different problems. To accommodate what seems like an impossible requirement, statistical classifications include “residual categories” at the bottom of the hierarchy, which fall into two types: Not Elsewhere Classified (no place else to put it) and Not Specified (unknown). These are often disparagingly referred to as wastebasket categories, but they serve a useful purpose in achieving exhaustiveness.

Since its inception in the late nineteenth century, the ICD has been and remains a statistical classification. Many terminology-oriented critics have pointed to its monohierarchy and residual categories as unacceptable failings (Cimino 1998); however, these are necessary elements of being a statistical classification.

## ICD-11

ICD-11 will succeed ICD-10 as the current version of the ICD managed by the World Health Organization (WHO) in June, 2018. ICD-10 was completed in 1990, making the interval between versions 28 years, the longest interval in the history of the ICD. Work began on ICD-11 in 2007, with the intention to make a more comprehensive and computable version of the ICD than previously available. As an aside, readers should be aware that many countries create an expanded version of the WHO ICD revision, such as the United States (ICD-10-CM), Australia (ICD-10-AM), Canada (ICD-10-CA), and Germany (ICD-10-GM). An aspiration is that ICD-11 many not require any country-specific extensions.

### Foundation component

A key architectural component of the ICD is the presence of a semantic network of words and terms, which forms the backbone of all statistical tabulations of the ICD-11 that derives from it. This the Foundation component, which is a large and deep polyhierarchy of medical concepts without any residual terms. The Foundation clearly violates the two conditions of a statistical classification, which remains the core use-case of the ICD; thus, a statistical tabulation must be created to accommodate the requirements of a statistical classification (below). However, the Foundation persists as a rich resource of terms, concepts, multiply parented relationships, and detail that is far in excess of what would be sensible in a statistical classification. As a practical matter, the Foundation semantic network has superseded the historical ICD index and become a basis for semi-automatic and algorithmic coding of terms into ICD rubrics.

Another novel feature of Foundation terms is that each has a small information model of globally unique identifiers (URIs), fully specified terms, definitions, synonyms, language variants, and many axial properties, such as options for anatomy, severity, extent, or etiology.

### Linearizations

To enable behavior as a statistical classification, ICD-11 draws a shallow monohierarchy tree onto the Foundation; for example, it determines which parent will prevail and how deep (or shallow) the hierarchy will be. This defines a derivative, called a linearization, of the Foundation that can be used as a statistical classification. The primary linearization, and the one most users will recognize and likely believe is “the ICD-11”, is the Mortality and Morbidity Statistics (MMS) linearization. This, of course, implies that one can have more than one linearized derivative, which is, in fact, the case. Work proceeds on a primary care linearization, and some subspecialty linearizations, such as Dermatology. Since all purpose-specific ICD linearizations derive from the same semantic network (the Foundation), they can all be algorithmically cross-walked and navigated.

Creating such linearizations also has implications. The most obvious is what we call the shoreline, where the Foundation may have depth of a score or more of layered categories, the MMS more typically has four, five, or, in some extreme cases, six layers of depth. This implies that substantial detail, such as highly specific or rare subtypes of disease, will be “below the shoreline” and relegated to a residual category in the MMS, even though they exist as a rubric in the Foundation. This is why many consider the Foundation as an index, though it is substantially more than simply that.

### Post-coordination

A new or expanded feature of the MMS in ICD-11 is the introduction of post-coordination, where two or more terms are combined into a “cluster” to more completely describe a patient condition. The implications here are two-fold: (1) the MMS is smaller than previous versions of the ICD, as many previously pre-coordinated terms are now below the shoreline in the classification. The second is that ICD-11 MMS is substantially more expressive and complete than previous versions, though this requires the invocation of multiple terms ensemble.

ICD-10, for example, had pre-coordinated colon cancer into specific anatomic sites of the colon (transverse, ascending, caecum, etc.). These pre-coordinated anatomic terms are eliminated from ICD-11 MMS, though they persist in the Foundation. However, they can be expressed by post-coordinating colon cancer with anatomic modifiers, which exist in a new chapter of modifiers. Correspondingly, one can also post-coordinate clinical stage, laterality, acuity, or, in some cases, germline genomic predispositions. The ability to compose a cluster of concepts within ICD-11 greatly extends its expressive power.

### Ontology connections

The design of ICD-11 incorporated a robust semantic anchoring of the Foundation through linkage to well-formed, formal ontologies, such as SNOMED CT, HGNC, and possibly HPO. While a compelling prototype, this work was never completed for resource reasons; nevertheless, it remains a body of work that may be advanced after the formal release of the MMS linearization in 2018.

A detailed technical description of the ICD-11 architecture and underlying informatics structure is in preparation and beyond the scope of the present manuscript.

## Implications for phenotype representation

ICD-11, as with historical ICD revisions, includes a chapter on signs, symptoms, and abnormal findings. This chapter is highly enriched with what many could consider the root of phenotyping features; however, disease signs and symptoms are emphasized more than rare anomalies. A deeply enriched chapter on developmental anomalies, expanded from the ICD-10 chapter on congenital malformations, is informed by contributions from the rare disease community and Orphanet in particular. There are also subchapter groupings, such as inborn errors of metabolism, which include specific phenotype manifestations.

ICD-11 heavily leverages post-coordination to describe anomalies in particular. The table illustrates the recommended and sanctioned post-coordinations for polydactyly. An example of validation post-coordination of polydactyly of the left intermediate phalanx of the hand is:

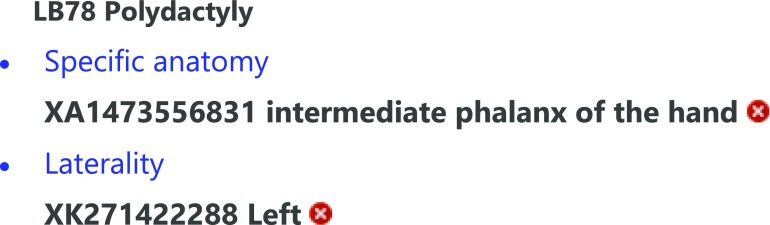



Combining into the ICD-11 MMS cluster code:

Code: LB78&XA1473556831&XK1694310660

Note that the “&” symbol is a syntactic convention which links codes into a single cluster. This introduces the use of base 34 numbers (0–9; A–Z except O and I) as MMS code values. Also, these code values may change by the June 2018 publication date, as these examples were drawn from a draft version of ICD-11 under review.

Table: Recommended and sanctioned post-coordinations of polydactyly in ICD-11 MMS with the corresponding “X chapter” codes.

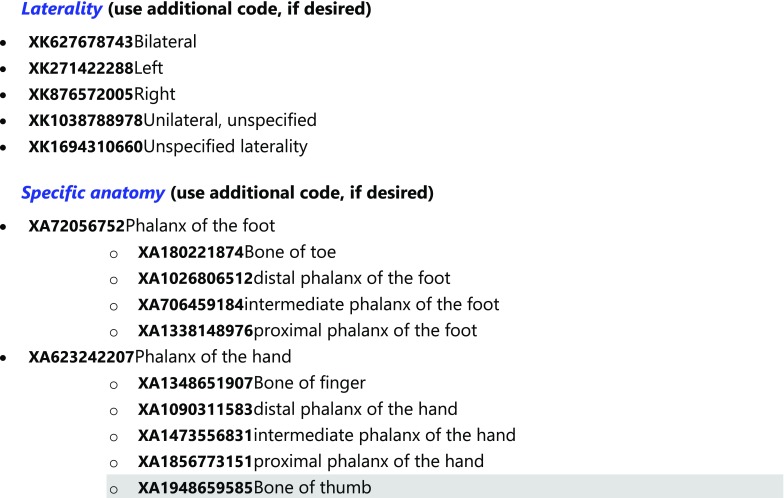



### Composite phenotype rendering

Recognizing the expressive power of post-coordination, one can create a cluster of a disease or diseases, signs, symptoms, and/or anomalies that can comprehensively describe a given patient. This aligns with the overloaded use of phenotyping as a description of a complex of findings to describe particular categories or subtypes of patients, as opposed to distinct features. ICD-11 is capable of representing these complexes as post-coordinated clusters of specific rubrics.

## The problem of rare diseases

Rare diseases present two problems in ICD-11: (1) sometimes appearing only in the Foundation and being below the shoreline of the MMS; and (2) raising questions as to which primary parent is most sensible in the MMS monohierarchy. Some rare diseases such as Lesch–Nyhan syndrome (5C35.01) illustrate that they keep their heads above the shoreline, though just barely with a rare six-digit code. However, while Fanconi anemia appears in the Foundation, it is relegated to a more general category: congenital aplastic anemia (3A70.0) exemplifying the first issue. Fanconi anemia does have a URI in the Foundation (http://id.who.int/icd/entity/1500851497), which is distinct from the base-34 code in the MMS. Further, while OMIM contains 19 entries in the phenotypic series for Fanconi anemia, the ICD-11 Foundation has only a single entry. In future, we expect expansion to the etiology and genomic modifier elements, which would allow a compositional expression of these 19 genomic variants of Fanconi anemia.

Substantial debate has occurred among the developers as to whether some hematological rare diseases belonged in the clinical hematology chapter or among the inborn errors of metabolism as a rare disease. In the Foundation, of course, they can be and are in both sections. In general, the legacy of ICD-10 would be determinative, though diseases that had no ICD-11 legacy tended to favor a clinically friendly category.

**Fig. 1:**
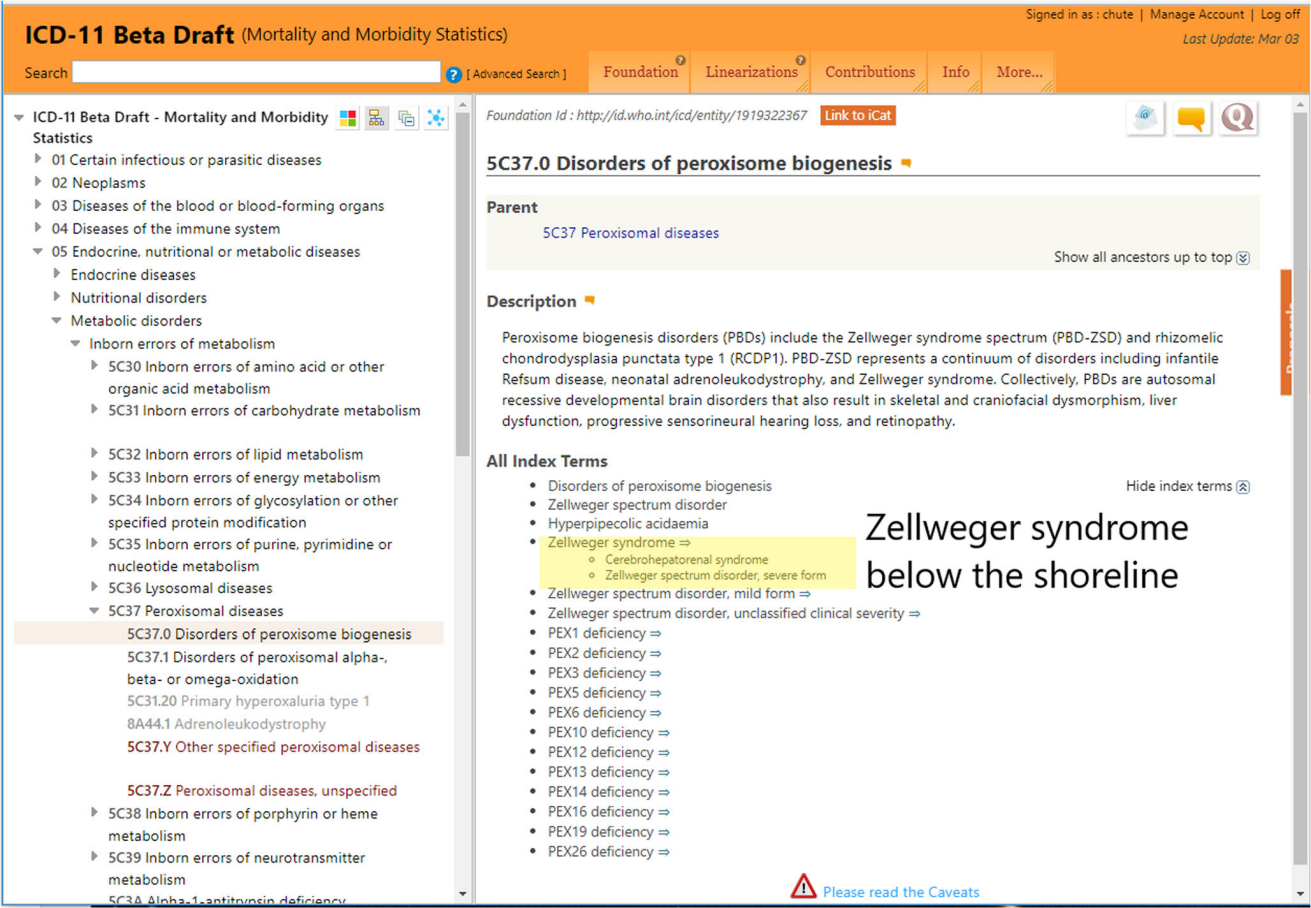
Zellweger syndrome is shown in the context of the Mortality and Morbidity Statistics (MMS) linearization, where it is below the shoreline and has no rubric codes. It is included only as an index entry

To expand upon these distinctions, Zellweger syndrome is illustrated in Fig. 1, which displays the WHO ICD-11 draft browser in the MMS mode. In the left column, hierarchical concepts cascade to detailed rubrics. In this version of the draft (03 March 2018), Zellweger syndrome is an index term below the shoreline beneath its primary linearization parent, 5C37.0 Disorders of peroxisome biogenesis. Note that the assigned rubric code, 5C37.0, is temporary and can vary from draft to draft; it will finalize with publication ICD-11 in the summer of 2018. In the right panel, Zellweger is highlighted in pale yellow among a score of index terms, none of which have specific rubric codes in the MMS and are, thus, by definition, below the shoreline.

**Fig. 2:**
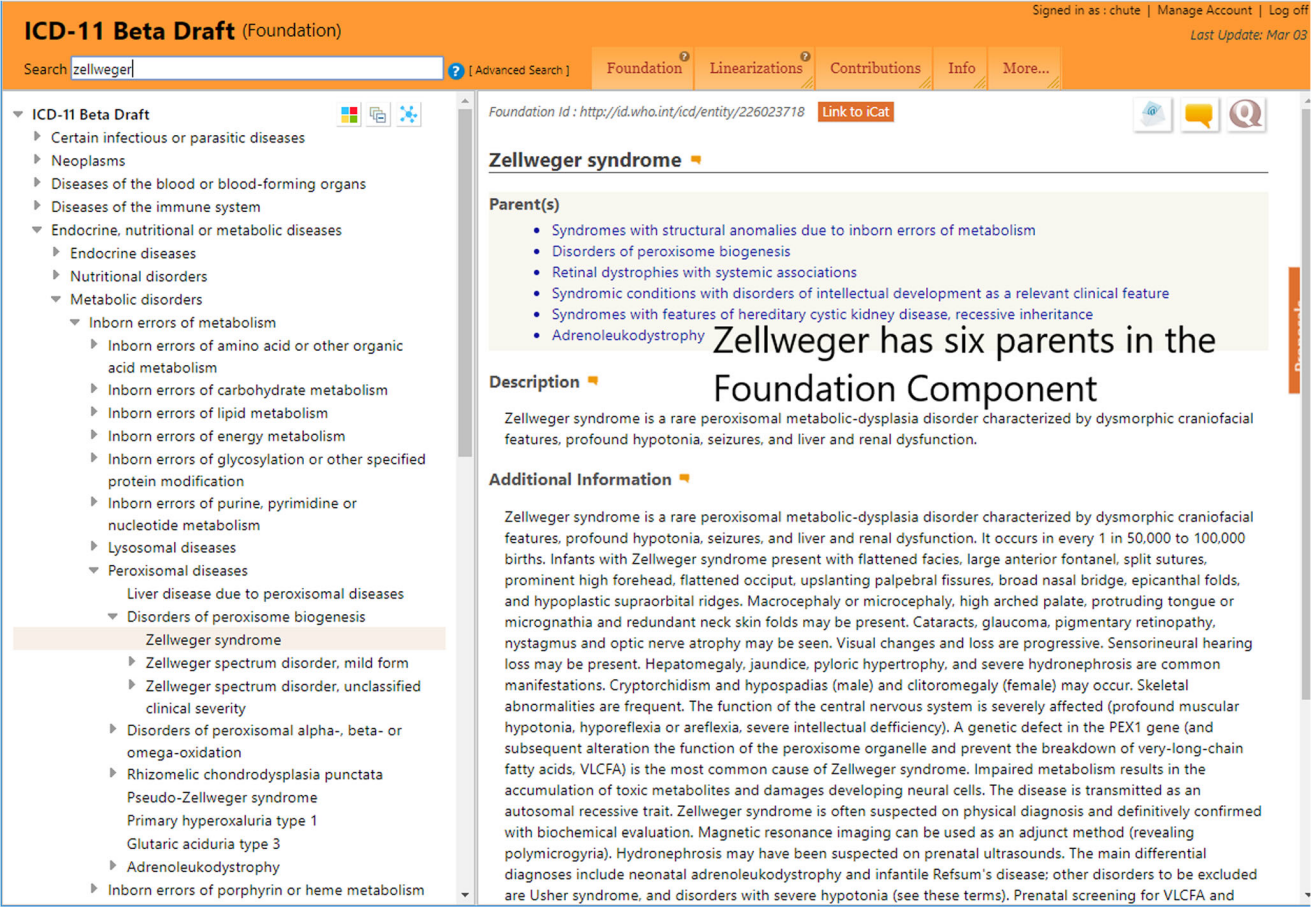
The same Zellweger syndrome appearing in the full Foundation component with its own identifier and descriptions. This entry has six parents, illustrating the polyhierarchy nature of the Foundation semantic network

Figure 2 illustrates the Foundation view of ICD-11, where Zellweger syndrome appears in the left panel beneath its primary MMS parent, Disorders of peroxisome biogenesis. However, Zellweger syndrome does have its own identifier, if not a traditional ICD rubric code, on the top of the right side: *Foundation Id*: http://id.who.int/icd/entity/1919322367 in gray. It is also obvious that Zellweger syndrome is a fully fledged Foundation entry, replete with its own content model, though only Parents, Description, and part of the Additional Information are shown. Notably, Zellweger syndrome has six parents in the Foundation, including its primary MMS parent. While not graphically conveyed, such multiple parenting illustrates the semantic network nature of the Foundation.

## Conclusion

ICD-11 (International Classification of Diseases, 11th Revision) is significantly more expressive and comprehensive than historical versions. The introduction of post-coordination makes the Mortality and Morbidity Statistics (MMS) linearization more compact, while allowing highly expressive compositional descriptions. Phenotype features are enriched, though fall short of purpose-specific systems such as HPO. Rare diseases existing in the Foundation are not always assigned a linearization code in the MMS, though they do have an independent URI.

Development of the ICD-11 Foundation and linkages to more robust ontologies will continue in the foreseeable future, which we expect will strengthen the use and application of the MMS linearization in clinical description.
